# *Bifidobacterium bifidum* Suppresses Gut Inflammation Caused by Repeated Antibiotic Disturbance Without Recovering Gut Microbiome Diversity in Mice

**DOI:** 10.3389/fmicb.2020.01349

**Published:** 2020-06-18

**Authors:** Miriam N. Ojima, Aina Gotoh, Hiromi Takada, Toshitaka Odamaki, Jin-Zhong Xiao, Toshihiko Katoh, Takane Katayama

**Affiliations:** ^1^Division of Integrated Life Science, Graduate School of Biostudies, Kyoto University, Kyoto, Japan; ^2^Next Generation Science Institute, Morinaga Milk Industry Co., Ltd., Zama, Japan

**Keywords:** gut microbiome, *Bifidobacterium bifidum*, probiotics, antibiotic disturbance, vancomycin

## Abstract

The gut microbiome is a dynamic community that significantly affects host health; it is frequently disturbed by medications such as antibiotics. Recently, probiotics have been proposed as a remedy for antibiotic-induced dysbiosis, but the efficacy of such treatments remains uncertain. Thus, the effect of specific antibiotic-probiotic combinations on the gut microbiome and host health warrants further research. We tested the effect vancomycin, amoxicillin, and ciprofloxacin on mice. Antibiotic administration was followed by one of the following recovery treatments: *Bifidobacterium bifidum* JCM 1254 as a probiotic (PR); fecal transplant (FT); or natural recovery (NR). Each antibiotic administration and recovery treatment was repeated three times over 9 weeks. We used the Shannon Index and Chao1 Index to determine gut microbiome diversity and assessed recovery by quantifying the magnitude of microbial shift using the Bray-Curtis Index of Dissimilarity. We determined the community composition by sequencing the V3–V4 regions of the 16S ribosomal RNA gene. To assess host health, we measured body weight and cecum weight, as well as mRNA expression of inflammation-related genes by reverse-transcription quantitative PCR. Our results show that community response varied by the type of antibiotic used, with vancomycin having the most significant effects. As a result, the effect of probiotics and fecal transplants also varied by antibiotic type. For vancomycin, the first antibiotic disturbance substantially increased the relative abundance of inflammatory species in the phylum Proteobacteria, such as *Proteus*, but the effect of subsequent disturbances was less pronounced, suggesting that the gut microbiome is affected by past disturbance events. Furthermore, although gut microbiome diversity did not recover, probiotic supplementation was effective in limiting cecum size enlargement and colonic inflammation caused by vancomycin. However, for amoxicillin and ciprofloxacin, the relative abundances of proinflammatory species were not greatly affected, and consequently, the effect of probiotic supplementation on community structure, cecum weight, and expression of inflammation-related genes was comparatively negligible. These results indicate that probiotic supplementation is effective, but only when antibiotics cause proinflammatory species-induced gut inflammation, suggesting that the necessity of probiotic supplementation is strongly influenced by the type of disturbance introduced to the community.

## Introduction

The ecological balance maintained by the gut microbial community is significant in establishing and maintaining host health. Previous studies show strong relationships between the gut microbiome and the host’s metabolism (reviewed by Rowland et al., 2018), nutrition (Yatsunenko et al., 2012), and immune function (Round and Mazmanian, 2009; Kau et al., 2011). On the other hand, dysbiosis, or a disturbance in the healthy microbiome, is linked to a variety of health issues such as obesity (Ley et al., 2005), diabetes (Qin et al., 2012; Kostic et al., 2015), asthma (Stokholm et al., 2018), and inflammatory bowel disease (IBD) (Petersen and Round, 2014). Though relatively stable over time (Faith et al., 2013), the gut microbiome experiences frequent disturbance, and the long term effects of repeated disturbance remain relatively understudied.

Disturbances to the gut microbiome can be caused by events such as the consumption of a high-fat diet (He et al., 2018), jet lag (Thaiss et al., 2014), and use of medications, especially antibiotics (Theriot et al., 2014). While antibiotics are important in combating diseases caused by pathogenic bacteria, they not only affect the target pathogen, but also the other beneficial and commensal species in the gut (Jernberg et al., 2007). Overuse of antibiotics can also lead to major clinical problems, such as the emergence of antibiotic-resistant strains (Levy and Marshall, 2004), weight gain (Cho et al., 2012; Gerber et al., 2016) and antibiotic-associated diarrhea (Hogenauer et al., 1998; Wiström et al., 2001; Elseviers et al., 2015). Furthermore, repeated antibiotic use has been reported to alter the composition of the gut microbiome long term (Dethlefsen and Relman, 2011).

Recent studies have suggested that the use of probiotics, or live microbes exogenously administered for therapeutic purposes, is a promising remedy for antibiotic-induced dysbiosis (Korpela et al., 2016; Ekmekciu et al., 2017). Probiotics have become increasingly popular — with a compound annual growth rate (CAGR) of 7.0%, the global probiotics market is expected to reach 63 billion USD by 2023 (Global Market Insights, 2016). However, the efficacy of such probiotic remedies remains debated, as many probiotic strains do not remain in the gut long term and are usually shed within 1–2 weeks (reviewed by Suez et al., 2019). Furthermore, a recent study suggested that probiotics may inhibit, rather than promote, recovery, while autologous fecal microbiome transplants were more effective (Suez et al., 2018).

Fecal microbiome transplants (FMT) have been used as a treatment for severe antibiotic-induced dysbiosis (Shahinas et al., 2012) and provide relatively rapid recovery from dysbiosis (Suez et al., 2018). However, despite increasing reports of successful treatments, the methodology is unstandardized (Goldenberg et al., 2018), and challenges for clinical implementation remain. Furthermore, several side effects, such as weight gain and diarrhea, have been reported (Alang and Kelly, 2015). In 2019, a death from an infection caused by *Escherichia coli* strains that produce extended-spectrum β-lactamase (ESBL) after FMT was reported (U.S. Food and Drug Administration, 2019). While recent studies on both probiotics and FMT suggest a therapeutic potential for microbiome-based treatments, studies often report conflicting results, indicating a need for further research.

One of the difficulties with probiotics research is the variety of probiotic strains available, leading to variability in reported results. For example, species in the genus *Bifidobacterium* are often used in probiotic therapies, but purported effects can vary not only at the species level but also at the strain level. When formula-fed infants were given either *Bifidobacterium longum* subspecies *infantis* (*B. infantis*) or *Bifidobacterium animalis* subspecies *lactis*, *B. infantis* was more effective in increasing fecal bifidobacteria and decreasing γ-Proteobacteria due to its superior ability to colonize the infant gut (Underwood et al., 2013). In a study by Gotoh et al. (2018), the addition of different *Bifidobacterium bifidum* strains to fecal cultures increased fecal bifidobacteria, but the ability of *B. bifidum* to increase the prevalence of other bifidobacterial species varied by strain (Katoh et al., 2020).

Many studies utilize a single combination of broad-spectrum antibiotics and pre-made probiotic blends; thus, the effect of specific antibiotic-probiotic combinations remains relatively understudied. The type, intensity, and frequency of disturbance is an important factor that shapes ecological communities and their response to subsequent recovery treatments. Therefore, we introduced a repeated disturbance to the gut microbiome with three types of antibiotics that have different bacterial targets and modes of action: vancomycin, amoxicillin, and ciprofloxacin. As a probiotic, we used *Bifidobacterium bifidum* JCM 1254, an infant-gut associated, altruistic species that extracellularly degrades complex sugars, such as human milk oligosaccharides (HMOs) and mucin *O*-glycans (Gotoh et al., 2018; Katoh et al., 2020). We present here a comparative analysis of the repeated antibiotic disturbance on the gut microbiome and the effect of probiotics on recovery in a lab-controlled experiment using mouse models.

## Materials and Methods

### Animals and Housing

We purchased 40 female C57BL/6 mice from Japan SLC, Inc. (Shizuoka, Japan) at 8–10 weeks of age. Mice were housed individually in polycarbonate cages with bedding and given free access to drinking water and a basal diet, Oriental MF (Oriental Yeast Co., Ltd., Tokyo, Japan), under controlled conditions of humidity (70%), lighting (12-h light/dark cycle), and temperature (22°C). The experiment began after a 2-week acclimation period. The protocols of the experiment were approved by the Kyoto University Animal Experimentation Committee (Lif-K18009 and Lif-K19022). Animal experiments were performed from August 21, 2018, to June 17, 2019.

### Antibiotics

Three types of antibiotics, vancomycin hydrochloride (Nacalai Tesque Inc., Kyoto, Japan), amoxicillin (LKT Laboratories, Inc., Minnesota, United States), and ciprofloxacin (LKT Laboratories, Inc., Minnesota, United States) were administered in drinking water for mice to ingest *ad libitum*. Concentrations of each antibiotic were calculated and adjusted for mice based on human dosages suggested by the US Food and Drug Administration (GlaxoSmithKline, 2006; Baxter Healthcare, 2007; Bayer HealthCare, 2017). We selected these antibiotics for their varied spectrum of activity and reported effects on the gut microbiome (Table 1).

**TABLE 1:** Summary of antibiotics used in the experiment.

**Antibiotic**	**Class**	**Bacterial target**	**Mode of action**	**Dosage**
Amoxicillin	Penicillin	Moderate spectrum	Inhibition of cell wall biosynthesis	0.22 mg/mL
Ciprofloxacin	Fluoroquinolone	Broad-spectrum, Gram-negatives	Inhibition of DNA replication	0.19 mg/mL
Vancomycin	Glycopeptide	Gram-positives	Inhibition of peptidoglycan synthesis	0.25 mg/mL

### Experimental Design

**FIGURE 1:**
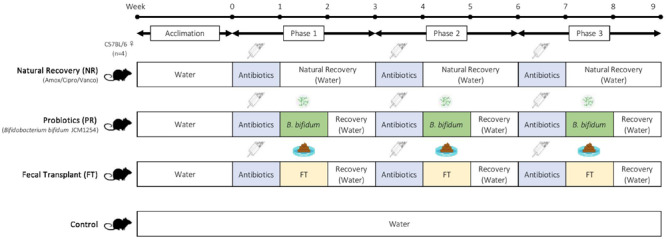
Experimental design. Adult female C57BL/6 mice were used in this experiment, with four biological replicates per group. After 2 weeks of acclimation, mice were given one of the following antibiotics for one week: amoxicillin, ciprofloxacin, or vancomycin. Antibiotic treatment was followed by one of the following recovery treatments: natural recovery (NR); *Bifidobacterium bifidum* JCM1254 as a probiotic (PR); and fecal transplant (FT). The recovery treatment was followed by 7 days of no treatments to allow the mice to recover. Each 3-week cycle was repeated three times during this 9-week experiment. Mice in the control were provided with water alone throughout the experiment.

Mice were divided into 10 groups (Table 2), with one control group and nine different antibiotic-recovery combinations, with four biological replicates per group. We determined the sample size based on power analyses and the resource equation approach (Arifin and Zahiruddin, 2017). Each group received antibiotics (vancomycin, amoxicillin, or ciprofloxacin) in drinking water for 7 days (antibiotics week). After antibiotics were administered, mice were switched to normal water without antibiotics and were given one of the following recovery treatments for 7 days (treatment week): natural recovery (NR); *Bifidobacterium bifidum* JCM 1254 as a probiotic (PR); or fecal transplant from control mice (FT). The treatment week was followed by 7 days with no treatments to allow the mice to recover (recovery week). During the treatment week and recovery week combined, we allowed the mice to recover from antibiotic administration for 14 days, as past studies have reported that the gut microbiome recovers within 1–2 weeks after disturbance (David et al., 2014; MacPherson et al., 2018). This was repeated three times for a total of three 3-week phases (Figure 1). Mice in the control were provided with water without antibiotics throughout the 9-week experiment.

**TABLE 2:** Treatment groups. Antibiotics were given to mice based on dosages described in Table 1.

**Treatment group**	**Antibiotics**	**Recovery**
Control		
A	Amoxicillin	Natural recovery (NR)
AB	Amoxicillin	*B. bifidum* JCM1254 (PR)
AF	Amoxicillin	Fecal transplant (FT; from control)
P	Ciprofloxacin	Natural recovery (NR)
PB	Ciprofloxacin	*B. bifidum* JCM1254 (PR)
PF	Ciprofloxacin	Fecal transplant (FT; from control)
V	Vancomycin	Natural recovery (NR)
VB	Vancomycin	*B. bifidum* JCM1254 (PR)
VF	Vancomycin	Fecal transplant (FT; from control)

*Natural recovery groups were administered PBS on each treatment day. Groups given probiotics as a recovery treatment were administered 10^9^ CFUs of *B. bifidum* per day. For fecal transplants, a mixture of fresh feces collected from age-matched control mice were suspended in PBS at a concentration of 40 mg/mL. After vortexing, the mixture was allowed to settle and the supernatant was administered.*

To prepare for probiotic administration, Gifu Anaerobic Medium (GAM, Nissui Pharmaceutical, Tokyo, Japan) was inoculated with *B. bifidum* each day, from glycerol stocks stored at −80°C, and incubated at 37°C overnight. From the overnight cultures, bacterial suspensions were diluted in phosphate-buffered saline (PBS) at a concentration of 10^9^ CFU per 200 μL. We then administered 200 μL of the bacterial suspensions to each mouse via oral gavage daily during the treatment weeks. For fecal transplants, a mixture of fresh feces collected from age-matched control mice were suspended in PBS at a concentration of 40 mg/mL and vortexed for 3 min. We then allowed the mixture to settle, and 200 μL of the supernatant was given to the mice via oral gavage daily during the treatment weeks. Mice in the control and NR groups were given 200 μL of PBS via oral gavage daily during the treatment weeks.

We also measured body weight as an indicator of feed intake and health. Fecal samples were collected from each mouse at the end of each week and stored at −30°C, and freeze-dried within a few days of collection. Freeze-dried fecal samples were stored at −30°C until use for DNA extraction. At the end of the experiment, animals were humanely euthanized by cervical dislocation. Immediately after death, a midline incision was made to exteriorize the intestine and cecum. Cecum weight was measured, and intestinal tissue samples were stored in RNA*later* (Invitrogen, Taastrup, Denmark) at 4°C until use.

### DNA Extraction

Freeze-dried fecal samples were placed in 2 mL plastic tubes with one stainless steel bead and approximately 200 mg of 0.1 mm zirconia beads and vigorously shaken for 10 min at 1500 rpm using the Shake Master NEO (Bio Medical Science, Tokyo, Japan) before extraction, as described previously (Sakanaka et al., 2019). Genomic DNA was extracted using a Qiagen QIAamp^®^ DNA Fast Stool Mini Kit (Hilden, Germany) according to the manufacturer’s instructions. Extracted DNA samples were stored at −30°C until use.

### Quantification of Total Bacterial Load Using Quantitative PCR

After genomic DNA extraction, we quantified the total bacterial load by measuring the number of copies of the 16S ribosomal RNA (16S rRNA) gene by quantitative PCR (qPCR) performed with a Thermal Cycler Dice Real-Time System (TaKaRa Bio., Kyoto, Japan). Each reaction mixture had a total volume of 15 μL and contained the following: 7.5 μL of TB Green^®^ Premix Ex Taq^TM^ II (TaKaRa Bio, Kyoto, Japan), 0.6 μL of each forward (5′-ACTCCTACGGGAGGCAGCAGT-3′) and reverse (5′-ATTACCGCGGCTGCTGGC-3′) primers, 1 μL of extracted DNA (diluted to 5 ng/μL), and 5.3 μL of water. The cycling conditions included an initial denaturation of 10 min at 95°C followed by 40 cycles of 95°C for 30 s and 68°C for 1 min. We used known concentrations of genomic DNA extracted from *Bacteroides thetaiotamicron* for reference curves for DNA quantification.

### Microbiome Analysis

Sequencing of the V3–V4 region of the 16S rRNA gene was performed with an Illumina MiSeq platform (Illumina, Inc., San Diego, CA, United States) as described previously (Odamaki et al., 2019). After removing sequences consistent with data from phiX reads from the raw Illumina paired-end reads, the sequences were analyzed using the QIIME2 software package version 2017.10^1^. After trimming of the 3′ region of the forward and the reverse reads (30 and 90 bases, respectively), the paired-end reads were joined, and potential chimeric sequences were removed using DADA2 (Callahan et al., 2016). Taxonomical classification was performed using a Naive Bayes classifier trained on the Greengenes 13.8 16S rRNA reference set with a 99% threshold of OTU full-length sequences. When possible, species were determined by Blastn analysis of the representative OTU sequences, for which the NCBI rRNA database was used.

### Quantification of Inflammation-Related Gene Expression Using Reverse-Transcription qPCR

Intestinal tissue samples were place in 2 mL plastic tubes with one stainless steel bead and approximately 200 mg of 0.1 mm zirconia beads. Samples were homogenized by vigorous shaking for 20 min at 1500 rpm using the Shake Master NEO (Bio Medical Science, Tokyo, Japan). Following RNA extraction using NucleoSpin^®^ RNA (TaKaRa Bio., Kyoto, Japan) according to the manufacturer’s instructions, cDNA was synthesized from 500 ng of total RNA by reverse transcription (RT) using PrimeScript II 1st strand cDNA Synthesis Kit (TaKaRa Bio., Kyoto, Japan). To measure the expression of inflammation-related genes in the intestinal tissue, RT-qPCR was carried out with a Thermal Cycler Dice Real-Time System (TaKaRa Bio). Each RT-qPCR reaction contained the following: 7.5 μL of TB Green^®^ Premix Ex Taq^TM^ II (TaKaRa Bio., Kyoto, Japan), 0.6 μL of each forward and reverse primers, 1 μL of the appropriately diluted cDNA solution, and 5.9 μL of water. The specificity of all primers was confirmed by analyzing the melting curves after the PCR was run. The cycling conditions were as follows: 95°C for 30 s, followed by 40 cycles of 95°C for 5 s, 60°C for 30 s, and a dissociation phase with 95°C for 15 s, 60°C for 30 s, and 95°C for 15 s. Standard curves were created for respective genes using the PCR-amplified fragments as templates. The primers were designed using Primer3 Plus software^2^, and the primer sets are listed in Supplementary Table 1.

### Diversity/Similarity Metrics and Statistical Analysis

Statistical analyses were performed using R ver. 3.6.0^3^. Species richness (α diversity) of the samples was estimated by the number of OTUs in each microbial profile using the Shannon Index (Shannon and Weaver, 1949) and the Chao1 Index. We used Two-Way Repeated Measures ANOVA (rm-ANOVA) with Tukey’s HSD *post hoc* test to determine the effect of each treatment over time. To determine the recovery of microbial communities, we quantified the magnitude of the microbial shift by comparing the microbiome profiles at baseline (Week 0) with profiles from other time points using the Bray-Curtis Dissimilarity Index. We further analyzed community structure using principal components analysis (PCA) and exploratory factor analysis. To determine the statistical differences in cecum weight and expression of inflammation-related genes, we used a One-Way ANOVA with *post hoc* Dunnett’s test. We also performed Pearson’s correlation analysis to identify specific taxa that were positively or negatively associated with cecum weight and expression of inflammation-related genes.

## Results and Discussion

The goal of this study was to assess the efficacy of the probiotic strain, *Bifidobacterium bifidum* JCM 1254, in the recovery period after the repeated antibiotic disturbance. Using mouse models, we administered three different antibiotics with varying bacterial targets and spectrum of activity. A subsequent recovery treatment consisted of *B. bifidum* supplementation or fecal transplants from healthy donor mice (age-matched mice from the control group). The key findings of this study are as follows: (1) the response of the gut microbiome varies significantly with the type of disturbance; (2) *B. bifidum* is most effective when antibiotic disturbance increases proinflammatory species; (3) probiotic supplementation does not restore the diversity of the gut microbiome to baseline levels but can contribute to the recovery of host health. Our results provide insight into how disturbance ecology affects the gut microbial community and its response to recovery treatments.

### Vancomycin Significantly Alters the Gut Microbiome and Increases Proinflammatory Species

We first compared the effect on the structure of the gut microbiome of repeated antibiotic exposure, testing vancomycin, ciprofloxacin, and amoxicillin (see Table 1 for spectrum and mode of action). To do so, we administered each antibiotic in drinking water for 7 days, allowed for 14 days of natural recovery, and repeated this process three times (Natural Recovery; Figure 1). Although statistically insignificant, the percent body weight increase had a tendency to be greater for all antibiotics compared to control (Supplementary Figures 1A–C). We then analyzed the fecal microbiome by meta-16S rRNA sequencing. For all antibiotic types, we did not see a significant variation in bacterial load over time (Supplementary Figures 1D–F). This is possibly because we collected fecal samples after seven days of antibiotic administration, which allowed the taxa unaffected by the antibiotics to proliferate during that time. Similar trends with vancomycin (Cheng et al., 2017) and amoxicillin (Cabral et al., 2019) have also been previously reported.

**FIGURE 2:**
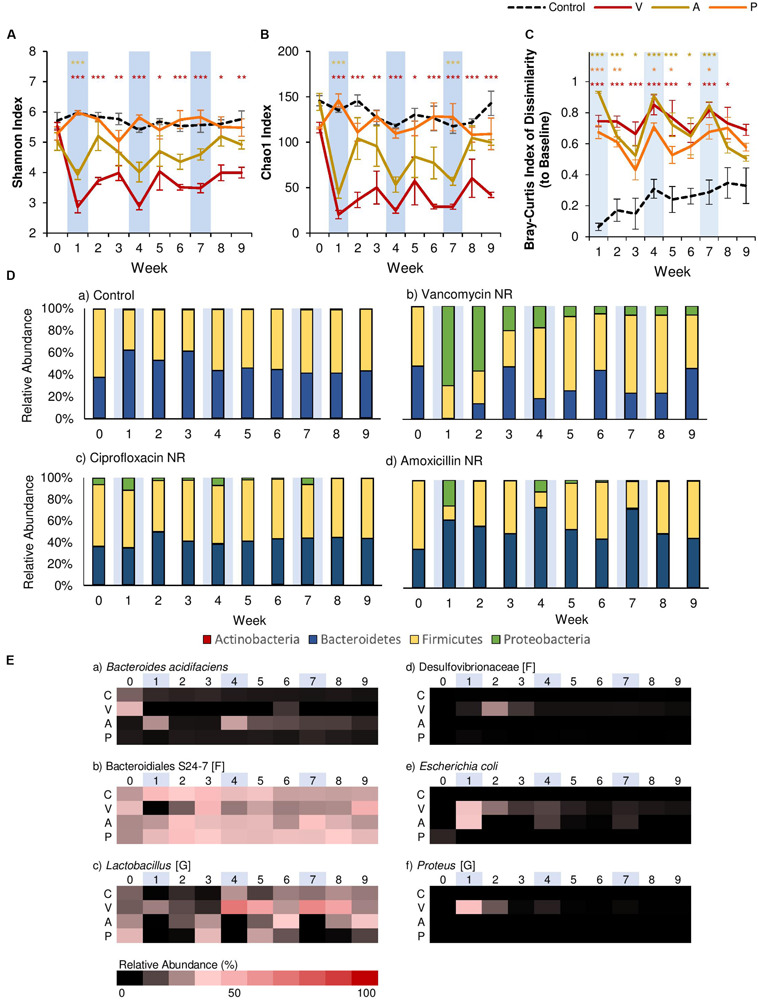
Comparison of the effects of vancomycin, ciprofloxacin, and amoxicillin on the gut microbiome. Each antibiotic was administered for 7 days every 3 weeks, and changes to the fecal microbiome over time were observed by meta-16S rRNA sequencing. Alpha diversity measured by **(A)** Shannon Index and **(B)** Chao1 Index for each treatment over time ± standard error. **(C)** Bray-Curtis Index of Dissimilarity vs baseline for each treatment over time ± standard error. The Bray-Curtis Index was used to quantify the amount of microbial shift from the first day of the experiment (baseline) for each individual. Colored asterisks indicate significance vs control for NR, PR, and FT groups based on Two-Way rm-ANOVA and Tukey’s HSD *post hoc* test (*p < 0.05, **p < 0.01, ***p < 0.001). Data for the control samples are indicated as the black dotted line. Natural recovery data for vancomycin is in red, amoxicillin in yellow, and ciprofloxacin in orange. **(D)** The microbial community at each time point at the phylum level for **(a)** control, **(b)** vancomycin, **(c)** ciprofloxacin, and **(d)** amoxicillin. **(E)** Heat map of taxa that significantly changed after antibiotic administration. Significant taxa were identified using factor analysis (factor loading >0.2). The lowest taxonomic rank for which information was available is indicated in square brackets (F: family, G: genus). Weeks shaded in blue indicate weeks in which antibiotics were administered.

However, clear differences between antibiotics were seen when we compared α-diversity using the Shannon Index (evenness; Figure 2A) and the Chao1 Index (species richness; Figure 2B). The results of Two-Way rm-ANOVA show that the type of antibiotic differentially affected α-diversity (Supplementary Table 2). For ciprofloxacin, antibiotic administration had no significant effect on α-diversity over time, even though ciprofloxacin has been shown to significantly alter the gut microbiome in human subjects (Dethlefsen and Relman, 2011). This may be because we utilized murine models, in which ciprofloxacin is shown to have a limited effect on the community structure of the gut microbiome in some studies (Schubert et al., 2015). Furthermore, ciprofloxacin is considered to have limited activity against anaerobic microbes (Goldstein and Citron, 1988). For amoxicillin, α-diversity was significantly reduced in terms of both evenness (>34% reduction) and species richness (>60% reduction) after the first antibiotic disturbance event, but recovered to control levels within two weeks. While this pattern continued after the second and third antibiotic disturbance events for species richness, evenness was not significantly affected after the first disturbance event, as amoxicillin is a β-lactam antibiotic that affects both Gram-positive and -negative bacteria. Of the three antibiotics, vancomycin had the strongest effect on α-diversity. The first antibiotic disturbance significantly reduced evenness (>52% reduction) and richness (>81% reduction), both of which did not recover throughout the experiment.

In addition to α-diversity, we assessed recovery by quantifying the magnitude of the microbial shift from baseline (Week 0) using the Bray-Curtis Index of Dissimilarity (Figure 2C), and the results of Two-Way rm-ANOVA show that the differences in the antibiotic type significantly affected the microbial communities during recovery (Supplementary Table 3). For this study, we considered communities that returned to baseline community structures based on this index as “recovered.” For both ciprofloxacin and amoxicillin, dissimilarity increased with each antibiotic disturbance event, and gradually decreased over the following two weeks. Examination at the phylum level showed that each antibiotic disturbance event increased the relative abundance of Proteobacteria (12%, ciprofloxacin; 20%, amoxicillin), which then decreased over time (Figure 2D). Although some level of recovery was observed, the microbial communities did not return to baseline levels throughout the experiment, which is consistent with previous studies that report that repeated antibiotic use leads to incomplete recovery (Dethlefsen and Relman, 2011). With vancomycin, the microbial communities displayed patterns consistent with α-diversity, and community dissimilarity remained high throughout the experiment after the first antibiotic disturbance. At the phylum level, the relative abundance of Proteobacteria increased significantly after the first antibiotic disturbance event (70%). Although this increase was diminished after the second (19%) and third (8%) antibiotic disturbance events, the presence of Proteobacteria was persistent throughout the experiment (Figure 2D). For all antibiotics, the increase in Proteobacteria was less pronounced with repeated disturbances. Further examination with principal components analysis (PCA) based on the microbial community composition corroborated these observations. For vancomycin- and amoxicillin-treated groups, PCA revealed that communities after the first antibiotic administration formed a separate cluster (Supplementary Figures 2A,B). The subsequent second and third antibiotic treatments for vancomycin and amoxicillin clustered closer to the control communities, lending further evidence to the fact that the gut microbiome retains the memory of past disturbance events (Dethlefsen and Relman, 2011). For ciprofloxacin-treated groups, however, the different treatments did not create clear clusters (Supplementary Figure 2C).

Further examination using exploratory factor analysis showed that the increase in Proteobacteria can be attributed to *Escherichia coli* for all antibiotics (Figure 2E, Supplementary Figure 3 and Supplementary Table 4). However, for vancomycin, there were also increases in proinflammatory species associated with dysbiosis. For example, after the first antibiotic disturbance event, we noted an increase in *Proteus* (34.7%, relative abundance; Figure 2E, Supplementary Figure 3), a genus associated with the onset of colitis (Shin et al., 2015). There was also an increase in the abundance of Desulfovibrionaceae (Figure 2E, Supplementary Figure 3), a family of sulfate-reducing bacteria often associated with high-fat diets (Clarke et al., 2012) during Week 2 (a 350-fold increase compared to baseline). In disturbance ecology, the type of disturbance is a critical factor that determines which specific members of the community are selected for over the course of time (Relman, 2012), and our results indicate that gut microbiome responses vary significantly by antibiotic type, with vancomycin having the most detrimental effects. Previous studies have shown that vancomycin is a particularly potent antibiotic that significantly reduces gut microbiome diversity (Vrieze et al., 2014) and causes intestinal dysbiosis (Cheng et al., 2017). Therefore, we focused on vancomycin and how different treatments (fecal transplants or probiotic administration) could contribute to the recovery of the gut microbial community in the following sections.

### Fecal Transplants Restore Gut Microbiome Diversity

**FIGURE 3:**
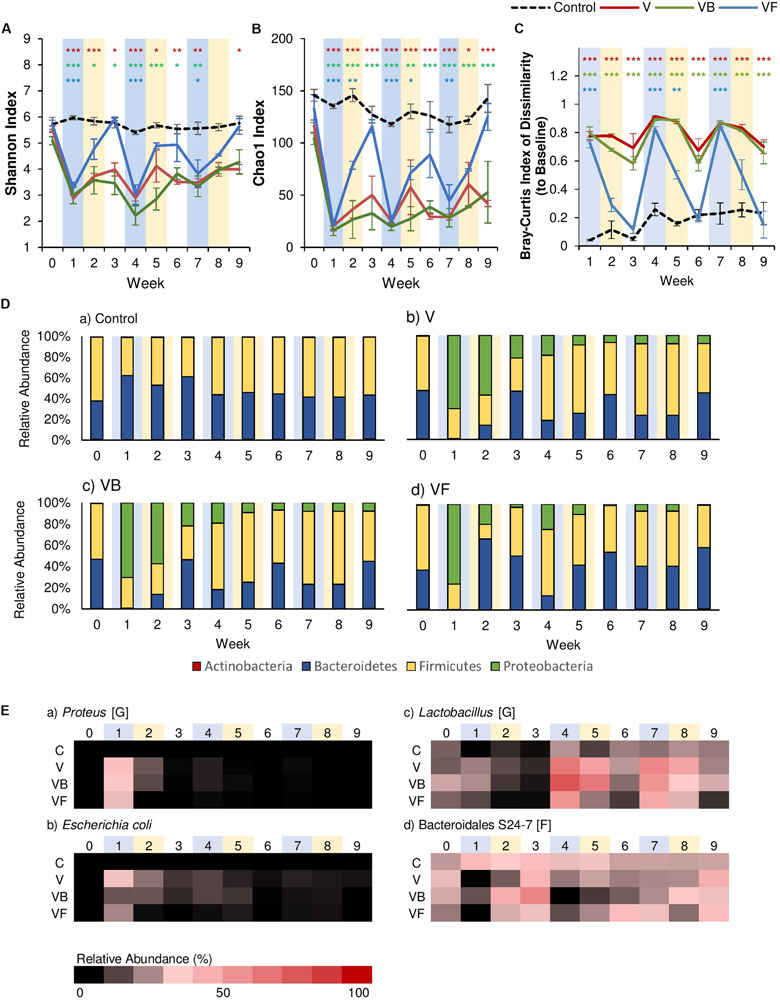
The effect of recovery treatments on the gut microbiome after vancomycin. Vancomycin administration was followed by either natural recovery (NR), *Bifidobacterium bifidum* (PR), or fecal transplants (FT), and changes to the gut microbiome were observed over time. Alpha diversity measured by **(A)** Shannon Index and **(B)** Chao1 Index for each treatment over time ± standard error. **(C)** Bray-Curtis Index of Dissimilarity vs baseline for each treatment over time ± standard error. The Bray-Curtis Index was used to quantify the amount of microbial shift from the first day of the experiment (baseline) for each individual. Colored asterisks indicate significance vs control for NR, PR, and FT groups based on Two-Way rm-ANOVA and Tukey’s HSD *post hoc* test (*p < 0.05, **p < 0.01, ***p < 0.001). Data for the control samples are indicated as the black dotted line, with NR (V) groups in red, PR (VB) groups in green, and FT (VF) groups in blue. **(D)** The microbial community at each time point at the phylum level for **(a)** control, **(b)** V, **(c)** VB, and **(d)** VF. **(E)** Heat map of taxa that significantly changed after antibiotic administration. Significant taxa were identified using factor analysis (factor loading >0.4). The lowest taxonomic rank for which information was available is indicated in square brackets (F: family, G: genus). Weeks shaded in blue indicate weeks in which antibiotics were administered, and weeks shaded in yellow indicate weeks in which a recovery treatment (natural recovery, probiotics, or fecal transplants) were administered.

Past studies have indicated that fecal transplants contribute to relatively rapid recovery after antibiotic-induced dysbiosis (Ekmekciu et al., 2017; Suez et al., 2018). After each vancomycin administration, we administered fecal transplants from healthy, age-matched control mice for seven days. As expected, the fecal transplants produced a significant effect on both α-diversity metrics, as well as community dissimilarity (Supplementary Tables 5, 6). α-Diversity was reduced after the first antibiotic disturbance event but completely recovered to control levels within two weeks of fecal transplants, and this pattern was observed for the subsequent disturbance events as well (Figures 3A,B). A similar pattern was observed for community dissimilarity, where each antibiotic administration increased dissimilarity, but fecal transplants restored community structures to baseline levels within two weeks (Figure 3C). Examination of community membership revealed that, compared to the natural recovery groups (V), fecal transplants were effective in reducing the Proteobacteria populations that had increased with each vancomycin administration (Figure 3D). While Proteobacteria populations persisted in the natural recovery groups (relative abundance >8%), Proteobacteria were nearly undetectable within two weeks after fecal transplants (relative abundance <1%), and the increase in inflammatory taxa such as *Proteus* was also suppressed (Figure 3E). Our results are consistent with previous studies, which have shown that fecal transplants are effective in correcting dysbiosis and reducing inflammation. Furthermore, a recent study by Burrello et al. (2018) demonstrated that fecal transplants promote recovery by stimulating immune cells to produce IL-10 and that the beneficial effects of fecal transplants seem to be correlated with the persistence of protective taxa such as Lactobacillaceae, Bifidobacteriaceae, Erysipelotrichaceae, Ruminococcacceae, and Bacteroidales S24-7.

### *Bifidobacterium bifidum* Does Not Restore Diversity but Reduces Intestinal Inflammation

In addition to fecal transplants, probiotic supplementation with species like *Bifidobacterium* in the gut has been linked to a variety of positive effects, such as reduced incidences of diarrhea in infants (Hotta et al., 1987), improvement in immune functions (Mohan et al., 2008), anti-obesity effects (Kondo et al., 2010; Stenman et al., 2014; Moya-Pérez et al., 2015), and recovery after antibiotic disturbance (Grazul et al., 2016; Ekmekciu et al., 2017). However, how effective probiotics are in restoring the disturbed gut microbial community after antibiotics remains a topic of debate (Suez et al., 2019). In our study, to assess the efficacy of probiotics in recovery after vancomycin administration, we administered *Bifidobacterium bifidum* JCM 1254 to mice via oral gavage for 7 days. Our results indicate that probiotic administration seemed to have little effect on recovery. Like the natural recovery groups, α-diversity did not return to baseline levels after the first antibiotic disturbance event (Figures 3A,B), and community dissimilarity remained high (Figure 3C). Similarly, Suez et al. (2018) also reported that a probiotic blend including *Lactobacillus, Bifidobacterium, Lactococcus*, and *Streptococcus* genera did not promote recovery after antibiotic-induced dysbiosis. These results suggest that species commonly called “probiotics” may be insufficient for community recovery.

Our exploratory factor analysis showed that the relative abundance of *Lactobacillus* species, *Proteus* species, *E. coli*, and Bacteroidales S24–7 contributed significantly to community structure (Supplementary Table 7). Although α-diversity did not recover, the first *B. bifidum* supplementation caused a two-fold increase in the relative abundance of Bacteroidales S24–7 (Figure 3E), a family of fermenters often associated with a healthy microbiome in mice that produce short-chain fatty acids (SCFA) and vitamin B (Evans et al., 2014; Rooks et al., 2014; Ormerod et al., 2016). While not as effective as fecal transplants, probiotics were also able to suppress the increase of Proteobacteria, such as *E. coli* and *Proteus* populations (Figure 3E). Previous studies have also reported the reduction of Proteobacteria after *Bifidobacterium* supplementation. For example, the administration of *Bifidobacterium longum* decreased the relative abundance of Proteobacteria and reduced the expression of the gene encoding TNF-α in mice (Lee et al., 2019), and *B. infantis* supplementation decreased γ-Proteobacteria in infants (Underwood et al., 2013).

**FIGURE 4:**
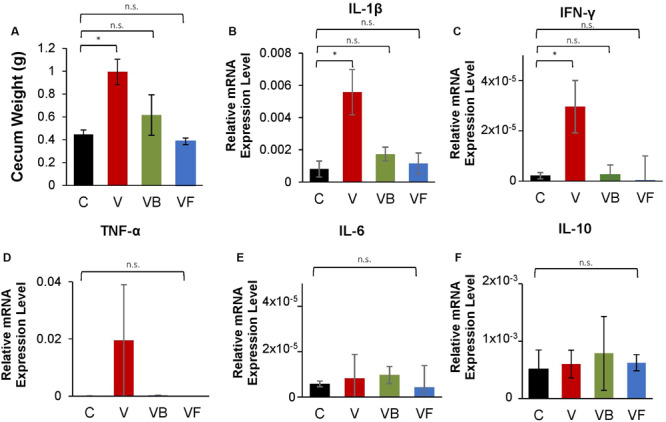
Changes in cecum size and expression of inflammation-related genes in vancomycin-treated mice. At the end of the experiment, we measured cecum weight and measured the mRNA expression levels of inflammation-related genes in the large intestine for vancomycin-treated mice (C: control, V: vancomycin + natural recovery, VB: vancomycin + *B*. *bifidum*, VF: vancomycin + fecal transplant from control mice). **(A)** Cecum weight, relative mRNA expression of genes encoding **(B)** IL-β, **(C)** TNF-α, **(D)** INF-γ, **(E)** IL-6, and **(F)** IL-10 for vancomycin-treated mice, using *Actb* as a reference gene. Error bars indicate standard error, and significance was determined by One-Way ANOVA and Dunnett’s test. (*p < 0.05, **p < 0.01, ***p < 0.001).

Furthermore, we observed that recovery treatments had a significant effect on cecum size (One-Way ANOVA: *F*(3,12) = 5.513, *p* < 0.05; Figure 4A). While mice in the natural recovery group (V) had a significantly larger cecum compared to the control (*post hoc* Dunnett’s test: *p* < 0.05), the difference was insignificant for groups given *B. bifidum* (VB) and fecal transplants (VF), suggesting that cecal enlargement was corrected by *B. bifidum* administration and fecal transplants. A previous study has also reported an enlargement in the cecum of antibiotic-treated mice, possibly because of a decrease in intestinal motility (Puhl et al., 2012). Cecal enlargement may also have been caused by the increase of pro-inflammatory species. Further analysis with Pearson’s correlation analysis revealed that there were strong positive correlations between cecum weight and the abundance of inflammatory taxa such as *Proteus* (*r* = 0.91, p < 0.001), and *E. coli* (*r* = 0.84, *p* < 0.001). Cecum weight and the expression of genes encoding IL-1β also showed a significant positive correlation (*r* = 0.70, *p* < 0.05).

Additionally, our RT-qPCR results indicate that the expression of genes encoding proinflammatory cytokines (IL-1β and INF-γ) was significantly increased in the natural recovery groups (Figures 4B,C), while expression was suppressed by probiotic administration and fecal transplants. Verma et al. (2019) recently identified *B. bifidum* cell surface polysaccharides as a factor that suppresses inflammation in the gut. Another possible anti-inflammatory mechanism may be modulated by indole-3-lactic acid (ILA) produced by *Bifidobacterium* species. ILA is an aromatic lactic acid and a metabolite of aromatic amino acids such as tryptophan, and serves as a ligand for the aryl hydrocarbon receptor (AhR) that regulates intestinal homeostasis. Meng et al. (2019) found that ILA produced by *B. infantis* had anti-inflammatory effects on infant enterocytes *in vitro*, and this metabolite is also reported to be produced by *B. bifidum* (Sakurai et al., 2019). A recent study by Laursen et al. (2020) has shown that *Bifidobacterium* species possess a specific enzyme that convert aromatic pyruvates, precursors of aromatic amino acids, into aromatic lactic acids. Given these results, we hypothesize that *B. bifidum* supplementation suppresses the increase of proinflammatory species and ultimately reduces gut inflammation.

We also examined the expression of genes encoding TNF-α, IL-6, and IL-10, but did not find any significant differences between treatments (Figures 4D–F), suggesting that antibiotic-induced dysbiosis leads to the induction of specific inflammatory cytokines. Furthermore, we repeated this experiment for amoxicillin- and ciprofloxacin-treated mice, testing administered fecal transplants and *B. bifidum*. However, the effects of neither fecal transplants nor probiotics differed significantly from NR groups for α-diversity and community structure (Supplementary Figures 4, 5), and no cecum enlargement was observed, even for antibiotic-treated groups (Supplementary Figures 6A, 7A). Furthermore, probiotics did not reduce the expression of inflammation-related genes after antibiotic administration. As neither amoxicillin nor ciprofloxacin caused a bloom in pro-inflammatory species (Supplementary Figures 4B–F, 5B–F), we hypothesize that in the absence of inflammatory species, the effect of fecal transplants and probiotic supplementation is negligible.

### Increase in *Lactobacillus* Abundance Potentially Delays Gut Microbiome Recovery

One common event we observed for all antibiotics was the expansion of *Lactobacillus*, particularly for vancomycin-treated groups (Figure 3E). We also observed that the increase was especially noticeable after the second antibiotic administration, which led us to believe that it may be due to the superior capability of *Lactobacillus* to tolerate disturbances. Past studies have shown *Lactobacillus* species to have a high level of vancomycin resistance (Gueimonde et al., 2013), as well as a relatively high tolerance to low pH (Corcoran et al., 2005; O’May et al., 2005). Furthermore, Suez et al. (2018) found that *Lactobacillus* was a microbiome-inhibitory species. Although unconfirmed in this study, it is possible that the increased relative abundance of *Lactobacillus* may have contributed to the inhibited recovery from antibiotic administration (Suez et al., 2018).

### Limitations

One of the limitations of this study is that we utilized a human-derived *Bifidobacterium* strain in murine models. Even in the human gut microbiome, the inability of probiotics to colonize the gut is a longstanding issue (Suez et al., 2019), but the lack of colonization was particularly evident in our study. Although we collected samples within 24 h of *B. bifidum* administration, its detection was limited in our 16S metagenomic analysis. Furthermore, we did not administer any prebiotics, possibly making colonization by *B. bifidum* in the gut even more difficult to achieve. A recent study by Cabral et al. (2019) has shown that the addition of fiber protected gut microbes from antibiotics, suggesting that the carbohydrates consumed in the diet alter the gut microbiome’s response to disturbances. Therefore, to develop more efficient probiotic therapies, future studies ought to consider the type of diet and prebiotics that are co-administered with antibiotics and probiotics.

### Conclusion

Despite these limitations, our study provides insight into how the gut microbiome responds to repeated disturbances and subsequent recovery treatments. In clinical settings, antibiotics are prescribed both frequently and repeatedly. A study based in the United Kingdom found that approximately 30% of patients are prescribed antibiotics at least once year (Shallcross et al., 2017). Although a different class of antibiotics is often re-prescribed with repeated use, results of our study elucidated how the repeated use of different types of antibiotics affects the response of the gut microbiome to recovery treatments. The type of disturbance (i.e., affected species, frequency, magnitude, and duration) is a key factor in community structuring, and its effect should be considered when examining the gut microbial community. The disturbance type determines which specific taxa and functions within the gut microbiome are selected for Relman (2012); moreover, we found that it also affects how the gut microbiome responds to the addition of probiotics. We found that probiotics were effective in reducing gut inflammation without recovering gut microbiome diversity. Additionally, our study showed that probiotics were most effective when antibiotic disturbance caused an increase in proinflammatory species. The results of the study could be applied to clinical settings, where predicting the response of the gut microbiome to different recovery treatments after dysbiosis would offer potential benefits.

## Data Availability Statement

The datasets generated for this study can be found in the EBI Metagenomics, under Accession Number PRJEB36500 (https://www.ebi.ac.uk/ena/browser/text-search?query=PRJEB36500).

## Ethics Statement

The animal study was reviewed and approved by Kyoto University Animal Experimentation Committee.

## Author Contributions

MO conceived the project. MO and AG designed the study and performed mouse experiments. HT performed the quantification of inflammation-related genes. TO and J-ZX contributed to the metagenome dataset analysis. MO analyzed the data, performed statistical analyses, and wrote and drafted the manuscript. All authors discussed the data and contributed to the completion of the final manuscript. TaK and ToK edited the manuscript. TaK supervised the study.

## Conflict of Interest

J-ZX and TO were employed by Morinaga Milk Industry Co., Ltd.

The remaining authors declare that the research was conducted in the absence of any commercial or financial relationships that could be construed as a potential conflict of interest.

## Footnotes

1 https://qiime2.org/

2 http://www.bioinformatics.nl/cgi-bin/primer3plus/primer3plus.cgi

3 www.r-project.org
