# Supplementary material for: Bifidobacterium bifidum Suppresses Gut Inflammation Caused by Repeated Antibiotic Disturbance Without Recovering Gut Microbiome Diversity in Mice
Source: Front Microbiol. 2020 Jun 18;11:1349. doi: 10.3389/fmicb.2020.01349 (PMC7314955; doi:10.3389/fmicb.2020.01349)
Supplement: Supplementary file 1 [file Data_Sheet_1.PDF]

Supplementary Figures

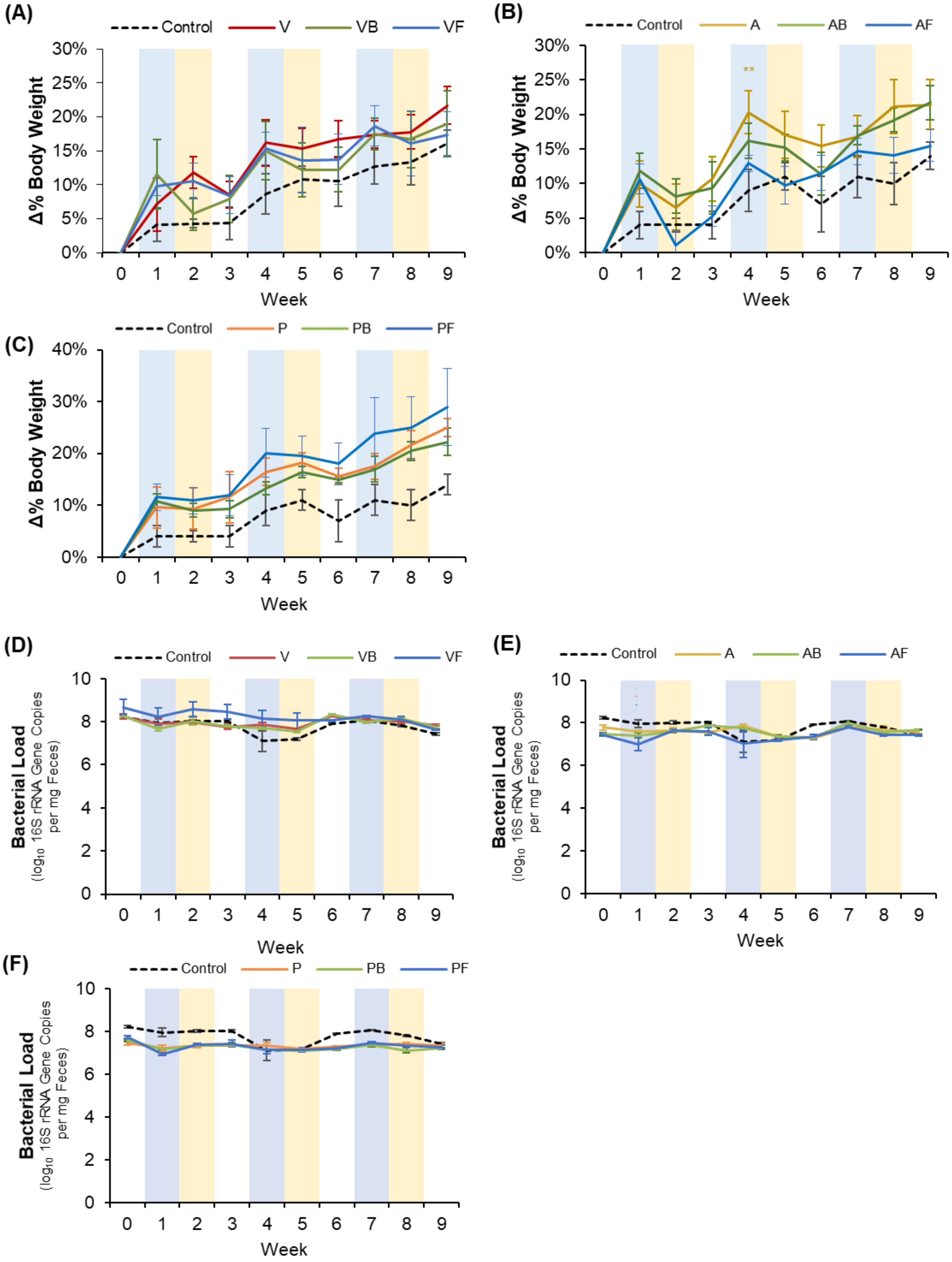

Supplementary Figure 1. Change in body weight and bacterial load over time.

Percent change in body weight over time for A) vancomycin, B) amoxicillin, and C) ciprofloxacin. Total bacterial load measured by the number of 16S rRNA gene copies per mg of feces using qPCR D) vancomycin, E) amoxicillin, and F) ciprofloxacin.
Weeks shaded in blue indicate weeks in which antibiotics were administered, and weeks shaded in yellow indicate weeks in which recovery treatments (natural recovery, probiotics, or fecal transplants) were administered.

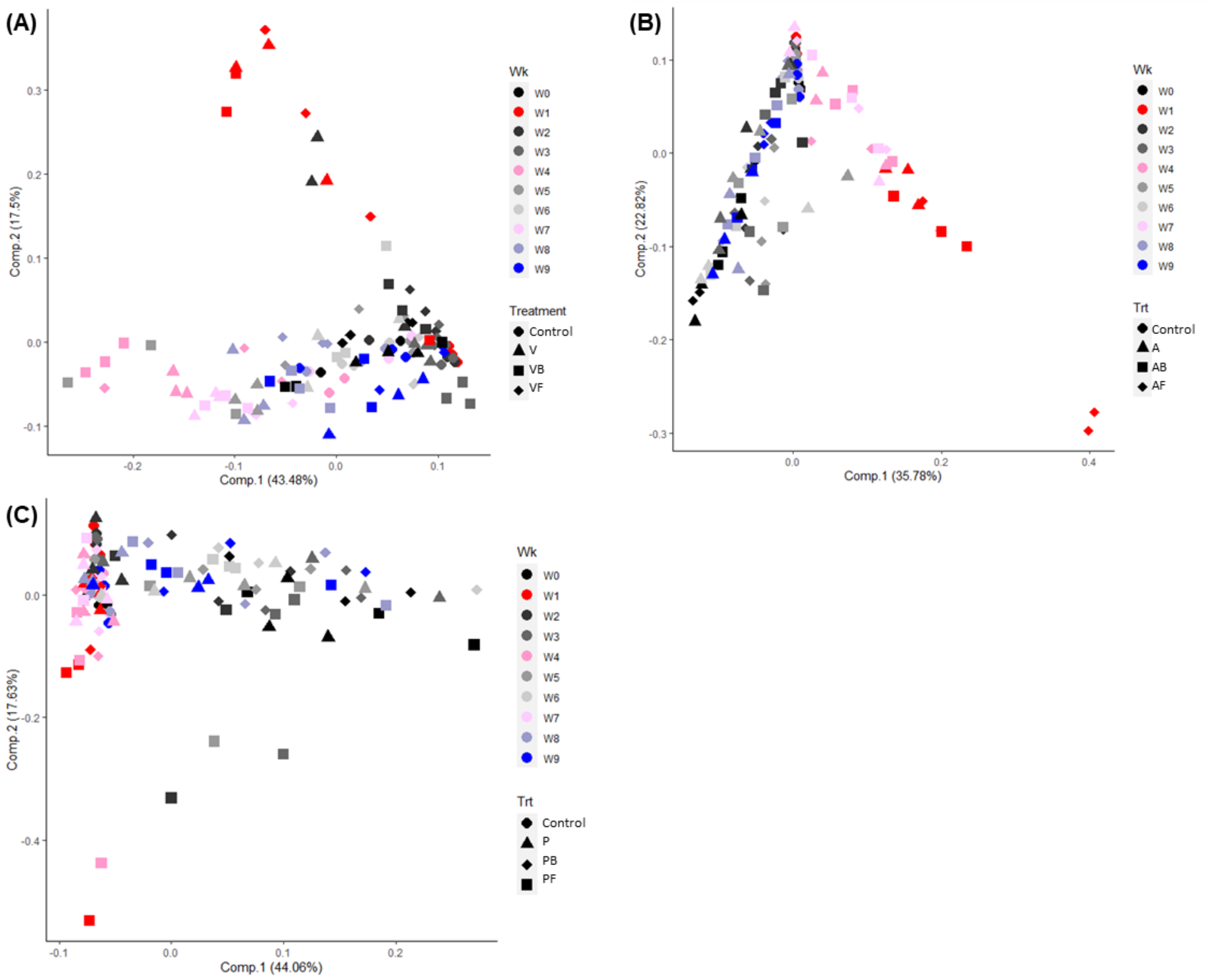

Supplementary Figure 2. Principal components analysis plot based on the microbial community composition.

Principal components analysis was performed based on microbial community composition for A) vancomycin-treated groups, B) amoxicillin treated groups, and C) ciprofloxacin treated groups. Treatment is denoted by different shapes (circle: control, triange: natural recovery, diamond: *B. bifidum*, square: fecal transplant) for each antibiotic group. Baseline communities (Week 0) are indicated in black, communities after the first antiboitc treatment are indicated in red, and the final communities are indicated in blue. The subsequent second and third antiboitc treatments are indicated in progressively lighter shades of red, and interim periods between antibiotic courses are indicated in progressively lighter shades of grey.

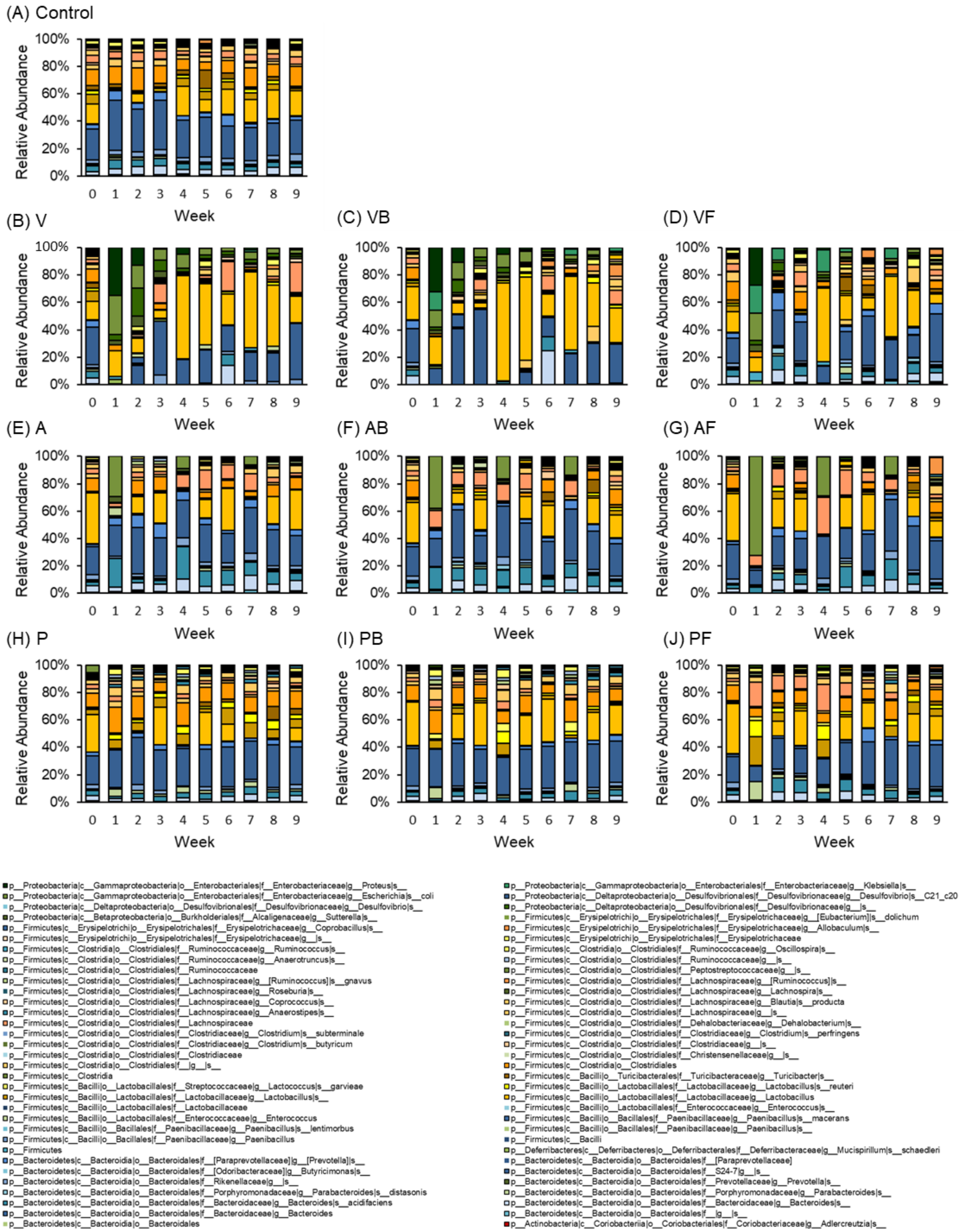
(A) Control
(B) V
(C) VB
(D) VF
(E) A
(F) AB
(G) AF
(H) P
(I) PB
(J) PF
Relative Abundance
100%
80%
60%
40%
20%
0%
0 1 2 3 4 5 6 7 8 9
Week
p__Proteobacteria|c__Gammaproteobacteria|o__Enterobacteriales|f__Enterobacteriaceae|g__Proteus|s__
p__Proteobacteria|c__Gammaproteobacteria|o__Enterobacteriales|f__Enterobacteriaceae|g__Escherichia|s__coli
p__Proteobacteria|c__Deltaproteobacteria|o__Desulfovibrionales|f__Desulfovibrionaceae|g__Desulfovibrio|s__
p__Proteobacteria|c__Betaproteobacteria|o__Burkholderiales|f__Alcaligenaceae|g__Sutterella|s__
p__Firmicutes|c__Erysipelotrichi|o__Erysipelotrichales|f__Erysipelotrichaceae|g__Coprobacillus|s__
p__Firmicutes|c__Erysipelotrichi|o__Erysipelotrichales|f__Erysipelotrichaceae|g__|s__
p__Firmicutes|c__Clostridia|o__Clostridiales|f__Ruminococcaceae|g__Ruminococcus|s__
p__Firmicutes|c__Clostridia|o__Clostridiales|f__Ruminococcaceae|g__Anaerotruncus|s__
p__Firmicutes|c__Clostridia|o__Clostridiales|f__Ruminococcaceae
p__Firmicutes|c__Clostridia|o__Clostridiales|f__Lachnospiraceae|g__[Ruminococcus]|s__gnavus
p__Firmicutes|c__Clostridia|o__Clostridiales|f__Lachnospiraceae|g__Roseburia|s__
p__Firmicutes|c__Clostridia|o__Clostridiales|f__Lachnospiraceae|g__Coprococcus|s__
p__Firmicutes|c__Clostridia|o__Clostridiales|f__Lachnospiraceae|g__Anaerostipes|s__
p__Firmicutes|c__Clostridia|o__Clostridiales|f__Lachnospiraceae
p__Firmicutes|c__Clostridia|o__Clostridiales|f__Clostridiaceae|g__Clostridium|s__subterminale
p__Firmicutes|c__Clostridia|o__Clostridiales|f__Clostridiaceae|g__Clostridium|s__butyricum
p__Firmicutes|c__Clostridia|o__Clostridiales|f__Clostridiaceae
p__Firmicutes|c__Clostridia|o__Clostridiales|f__|g__|s__
p__Firmicutes|c__Clostridia
p__Firmicutes|c__Bacilli|o__Lactobacillales|f__Streptococcaceae|g__Lactococcus|s__garvieae
p__Firmicutes|c__Bacilli|o__Lactobacillales|f__Lactobacillaceae|g__Lactobacillus|s__
p__Firmicutes|c__Bacilli|o__Lactobacillales|f__Lactobacillaceae
p__Firmicutes|c__Bacilli|o__Lactobacillales|f__Enterococcaceae|g__Enterococcus
p__Firmicutes|c__Bacilli|o__Bacillales|f__Paenibacillaceae|g__Paenibacillus|s__lentimorbus
p__Firmicutes|c__Bacilli|o__Bacillales|f__Paenibacillaceae|g__Paenibacillus
p__Firmicutes
p__Bacteroidetes|c__Bacteroidia|o__Bacteroidales|f__[Paraprevotellaceae]|g__[Prevotella]|s__
p__Bacteroidetes|c__Bacteroidia|o__Bacteroidales|f__[Odoribacteraceae]|g__Butyricimonas|s__
p__Bacteroidetes|c__Bacteroidia|o__Bacteroidales|f__Rikenellaceae|g__|s__
p__Bacteroidetes|c__Bacteroidia|o__Bacteroidales|f__Porphyromonadaceae|g__Parabacteroides|s__distasonis
p__Bacteroidetes|c__Bacteroidia|o__Bacteroidales|f__Bacteroidaceae|g__Bacteroides|s__acidifaciens
p__Bacteroidetes|c__Bacteroidia|o__Bacteroidales|f__Bacteroidaceae|g__Bacteroides
p__Bacteroidetes|c__Bacteroidia|o__Bacteroidales
p__Proteobacteria|c__Gammaproteobacteria|o__Enterobacteriales|f__Enterobacteriaceae|g__Klebsiella|s__
p__Proteobacteria|c__Deltaproteobacteria|o__Desulfovibrionales|f__Desulfovibrionaceae|g__Desulfovibrio|s__C21_c20
p__Proteobacteria|c__Deltaproteobacteria|o__Desulfovibrionales|f__Desulfovibrionaceae|g__|s__
p__Firmicutes|c__Erysipelotrichi|o__Erysipelotrichales|f__Erysipelotrichaceae|g__[Eubacterium]|s__dolichum
p__Firmicutes|c__Erysipelotrichi|o__Erysipelotrichales|f__Erysipelotrichaceae|g__Allobaculum|s__
p__Firmicutes|c__Erysipelotrichi|o__Erysipelotrichales|f__Erysipelotrichaceae
p__Firmicutes|c__Clostridia|o__Clostridiales|f__Ruminococcaceae|g__Oscillospira|s__
p__Firmicutes|c__Clostridia|o__Clostridiales|f__Ruminococcaceae|g__|s__
p__Firmicutes|c__Clostridia|o__Clostridiales|f__Peptostreptococcaceae|g__|s__
p__Firmicutes|c__Clostridia|o__Clostridiales|f__Lachnospiraceae|g__[Ruminococcus]|s__
p__Firmicutes|c__Clostridia|o__Clostridiales|f__Lachnospiraceae|g__Lachnospira|s__
p__Firmicutes|c__Clostridia|o__Clostridiales|f__Lachnospiraceae|g__Blautia|s__producta
p__Firmicutes|c__Clostridia|o__Clostridiales|f__Lachnospiraceae|g__|s__
p__Firmicutes|c__Clostridia|o__Clostridiales|f__Dehalobacteriaceae|g__Dehalobacterium|s__
p__Firmicutes|c__Clostridia|o__Clostridiales|f__Clostridiaceae|g__Clostridium|s__perfringens
p__Firmicutes|c__Clostridia|o__Clostridiales|f__Clostridiaceae|g__|s__
p__Firmicutes|c__Clostridia|o__Clostridiales|f__Christensenellaceae|g__|s__
p__Firmicutes|c__Clostridia|o__Clostridiales
p__Firmicutes|c__Bacilli|o__Turicibacterales|f__Turicibacteraceae|g__Turicibacter|s__
p__Firmicutes|c__Bacilli|o__Lactobacillales|f__Lactobacillaceae|g__Lactobacillus|s__reuteri
p__Firmicutes|c__Bacilli|o__Lactobacillales|f__Lactobacillaceae|g__Lactobacillus
p__Firmicutes|c__Bacilli|o__Lactobacillales|f__Enterococcaceae|g__Enterococcus|s__
p__Firmicutes|c__Bacilli|o__Bacillales|f__Paenibacillaceae|g__Paenibacillus|s__macerans
p__Firmicutes|c__Bacilli|o__Bacillales|f__Paenibacillaceae|g__Paenibacillus|s__
p__Firmicutes|c__Bacilli
p__Deferribacteres|c__Deferribacteres|o__Deferribacterales|f__Deferribacteraceae|g__Mucispirillum|s__schaedleri
p__Bacteroidetes|c__Bacteroidia|o__Bacteroidales|f__[Paraprevotellaceae]
p__Bacteroidetes|c__Bacteroidia|o__Bacteroidales|f__S24-7|g__|s__
p__Bacteroidetes|c__Bacteroidia|o__Bacteroidales|f__Prevotellaceae|g__Prevotella|s__
p__Bacteroidetes|c__Bacteroidia|o__Bacteroidales|f__Porphyromonadaceae|g__Parabacteroides|s__
p__Bacteroidetes|c__Bacteroidia|o__Bacteroidales|f__Bacteroidaceae|g__Bacteroides|s__
p__Bacteroidetes|c__Bacteroidia|o__Bacteroidales|f__|g__|s__
p__Actinobacteria|c__Coriobacteriia|o__Coriobacteriales|f__Coriobacteriaceae|g__Adlercreutzia|s__

Supplementary Figure 3. Microbial community composition based on sequencing data of the V3-V4 regions of the 16S rRNA gene.

The composition of the gut microbial community over time was determined based on sequencing data of the V3-V4 region of the 16S rRNA gene. For each treatment, relative abundance (%) of each taxa is shown (the lowest taxonomic rank for which information was available). A) C: control, B) V: vancomycin + natural recovery, C) VB: vancomycin + *B. bifidum*, D) VF: vancomycin + fecal transplant, E) A: amoxicillin + natural recovery, F) AB: amoxicillin + *B. bifidum*, G) AF: amoxicillin + fecal transplant, H) P: ciprofloxacin + natural recovery, I) PB: ciprofloxacin + *B. bifidum*, J) PF: ciprofloxacin + fecal transplant.

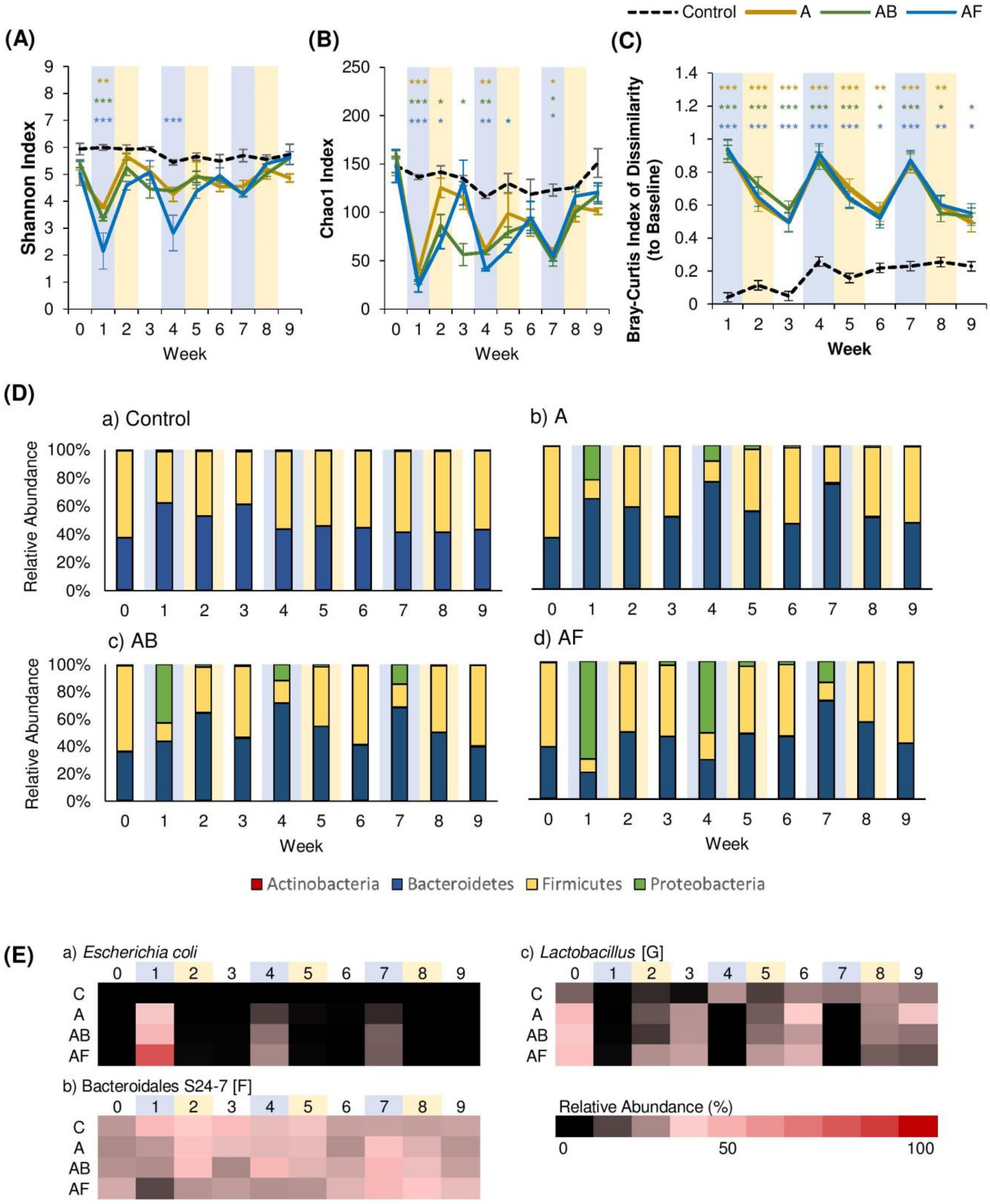
Control
A
AB
AF
(A)
Shannon Index
Week
(B)
Chao1 Index
Week
(C)
Bray-Curtis Index of Dissimilarity (to Baseline)
Week
(D)
a) Control
b) A
c) AB
d) AF
Relative Abundance
Week
Actinobacteria
Bacteroidetes
Firmicutes
Proteobacteria
(E)
a) Escherichia coli
b) Bacteroidales S24-7 [F]
c) Lactobacillus [G]
C
A
AB
AF
Relative Abundance (%)
0
50
100

Supplementary Figure 4. The effect of recovery treatments on the gut microbiome after amoxicillin.

Amoxicillin administration was followed by either natural recovery (NR), *Bifidobacterium bifidum* (PR), or fecal transplants (FT), and changes to the gut microbiome were observed over time.

Alpha diversity measured by A) Shannon Index and B) Chao1 Index for each treatment over time ± standard error. C) Bray-Curtis Index of Dissimilarity vs baseline for each treatment over time ± standard error. The Bray-Curtis Index was used to quantify the amount of microbial shift from the first day of the experiment (baseline) for each individual. Colored asterisks indicate significance vs control for NR, PR, and FT groups based on Two-Way rm-ANOVA and Tukey's HSD post hoc test (* $p < 0.05$, ** $p < 0.01$, *** $p < 0.001$). Data for the control samples are indicated as the black dotted line, with NR groups in red, PR groups in green, and FT groups in blue.

D) The microbial community at each time point at the phylum level for a) control, b) NR (A), c) PR (AB) and d) FT (AF). E) Heat map of taxa that significantly changed after antibiotic administration. Significant taxa were identified using factor analysis (factor loading >0.2). The lowest taxonomic rank for which information was available is indicated in square brackets (F: family, G: genus). Weeks shaded in blue indicate weeks in which antibiotics were administered, and weeks shaded in yellow indicate weeks in which recovery treatments (natural recovery, probiotics, or fecal transplants) were administered.

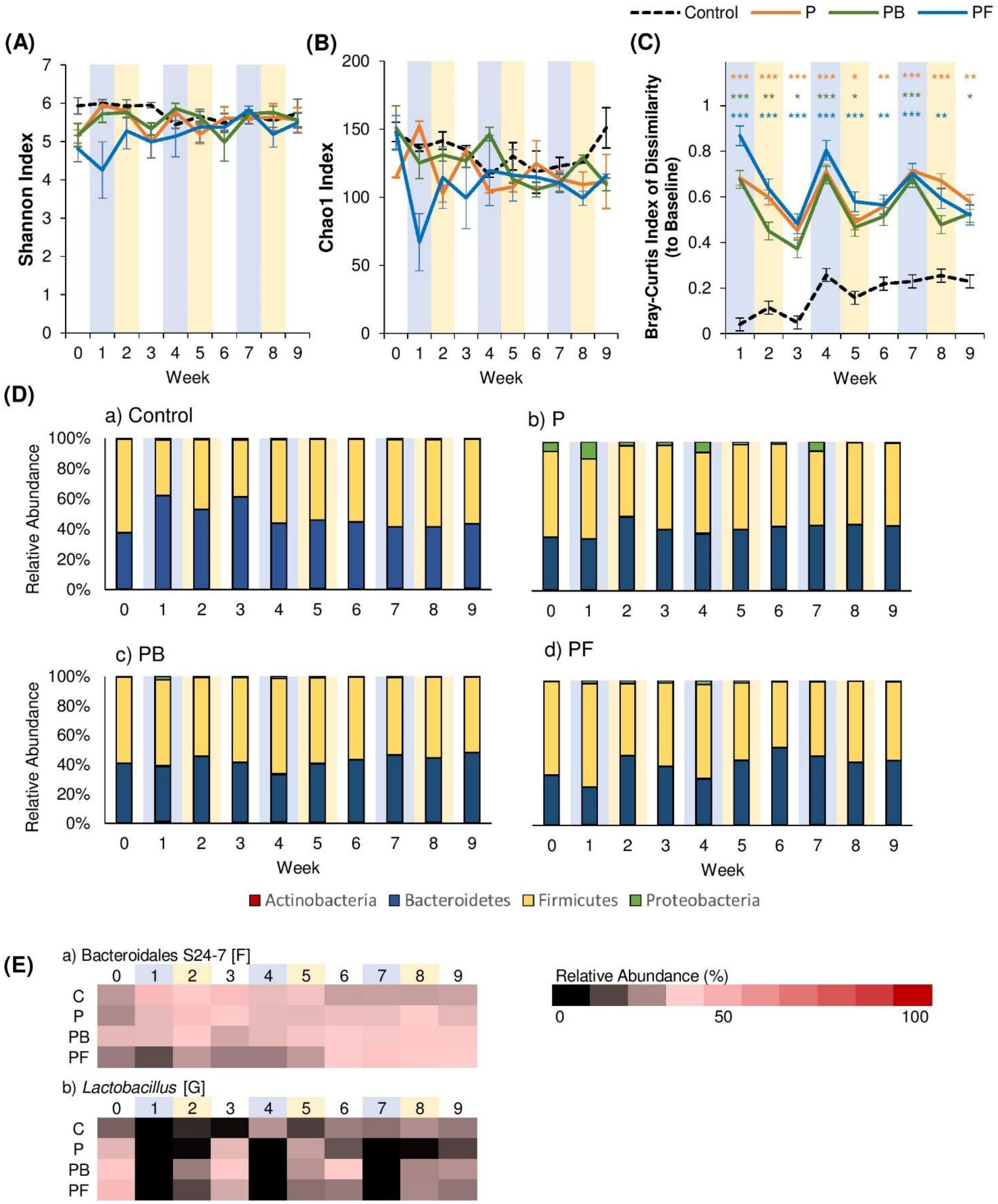
Control
P
PB
PF
(A)
Shannon Index
Week
(B)
Chao1 Index
Week
(C)
Bray-Curtis Index of Dissimilarity (to Baseline)
Week
(D)
a) Control
b) P
c) PB
d) PF
Relative Abundance
Week
Actinobacteria
Bacteroidetes
Firmicutes
Proteobacteria
(E)
a) Bacteroidales S24-7 [F]
b) Lactobacillus [G]
C
P
PB
PF
Relative Abundance (%)
0
50
100

Supplementary Figure 5. The effect of recovery treatments on the gut microbiome after ciprofloxacin.

Ciprofloxacin administration was followed by either natural recovery (NR), *Bifidobacterium bifidum* (PR), or fecal transplants (FT), and changes to the gut microbiome were observed over time.

Alpha diversity measured by A) Shannon Index and B) Chao1 Index for each treatment over time ± standard error. C) Bray-Curtis Index of Dissimilarity vs baseline for each treatment over time ± standard error. The Bray-Curtis Index was used to quantify the amount of microbial shift from the first day of the experiment (baseline) for each individual. Colored asterisks indicate significance vs control for NR, PR, and FT groups based on Two-Way rm-ANOVA and Tukey's HSD post hoc test (* $p < 0.05$, ** $p < 0.01$, *** $p < 0.001$). Data for the control samples are indicated as the black dotted line, with NR groups in red, PR groups in green, and FT groups in blue.

D) The microbial community at each time point at the phylum level for a) control, b) NR (P), c) PR (PB) and d) FT (PF). E) Heat map of taxa that significantly changed after antibiotic administration. Significant taxa were identified using factor analysis (factor loading >0.2). The lowest taxonomic rank for which information was available is indicated in square brackets (F: family, G: genus). Weeks shaded in blue indicate weeks in which antibiotics were administered, and weeks shaded in yellow indicate weeks in which recovery treatments (natural recovery, probiotics, or fecal transplants) were administered.

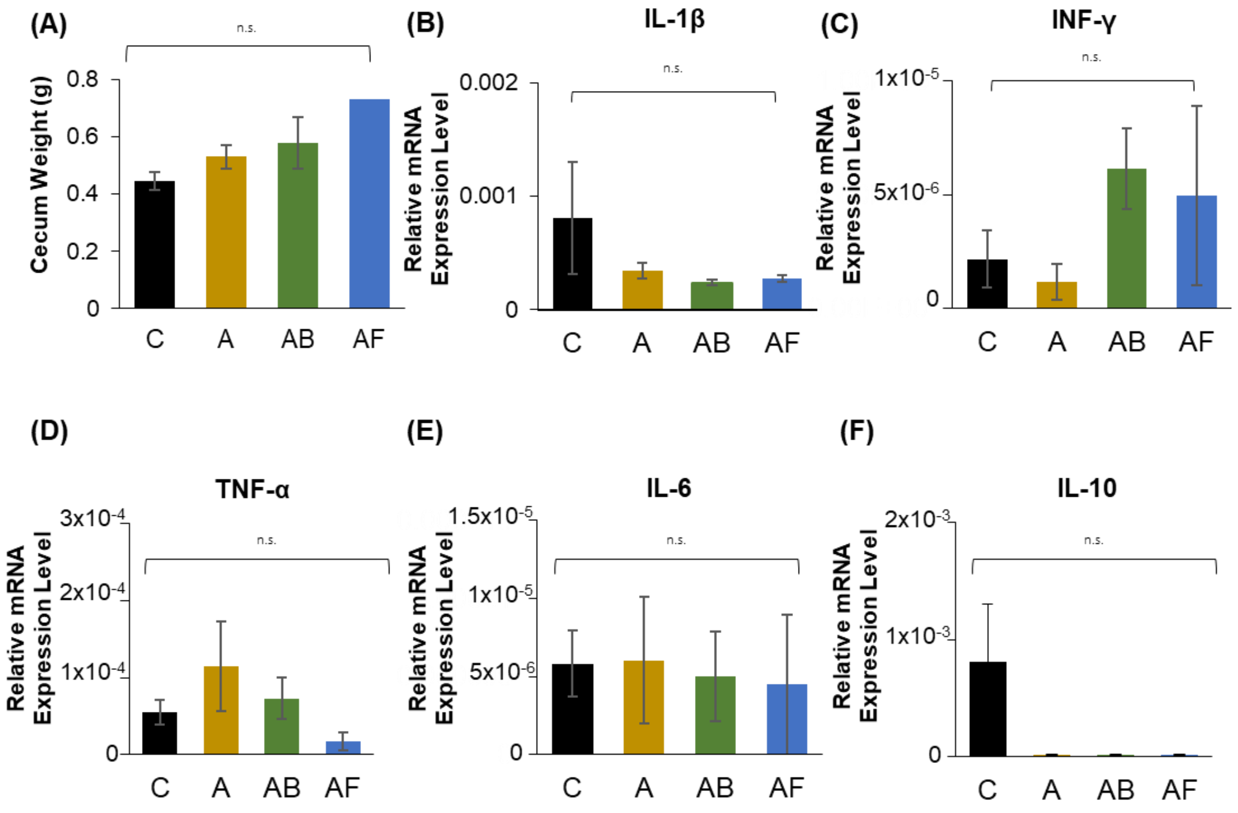

Supplementary Figure 6. Changes in cecum size and expression of inflammation-related genes in amoxicillin-treated mice.

At the end of the experiment, we measured cecum weight and measured the mRNA expression levels of inflammation-related genes in the large intestine for amoxicillin-treated mice (C: control, A: amoxicillin + natural recovey, AB: amoxicillin + *B. bifidum*, AF: amoxicillin + fecal transplant from control mice). A) Cecum weight, relative mRNA expression of genes encoding B) IL-β, C) TNF-α, D) INF-γ, E) IL-6, and F) IL-10 for amoxicillin-treated mice, using *Actb* as a reference gene. Error bars indicate standard error, and significance was determined by One-Way ANOVA and Dunnett's test. (* $p < 0.05$, ** $p < 0.01$, *** $p < 0.001$).

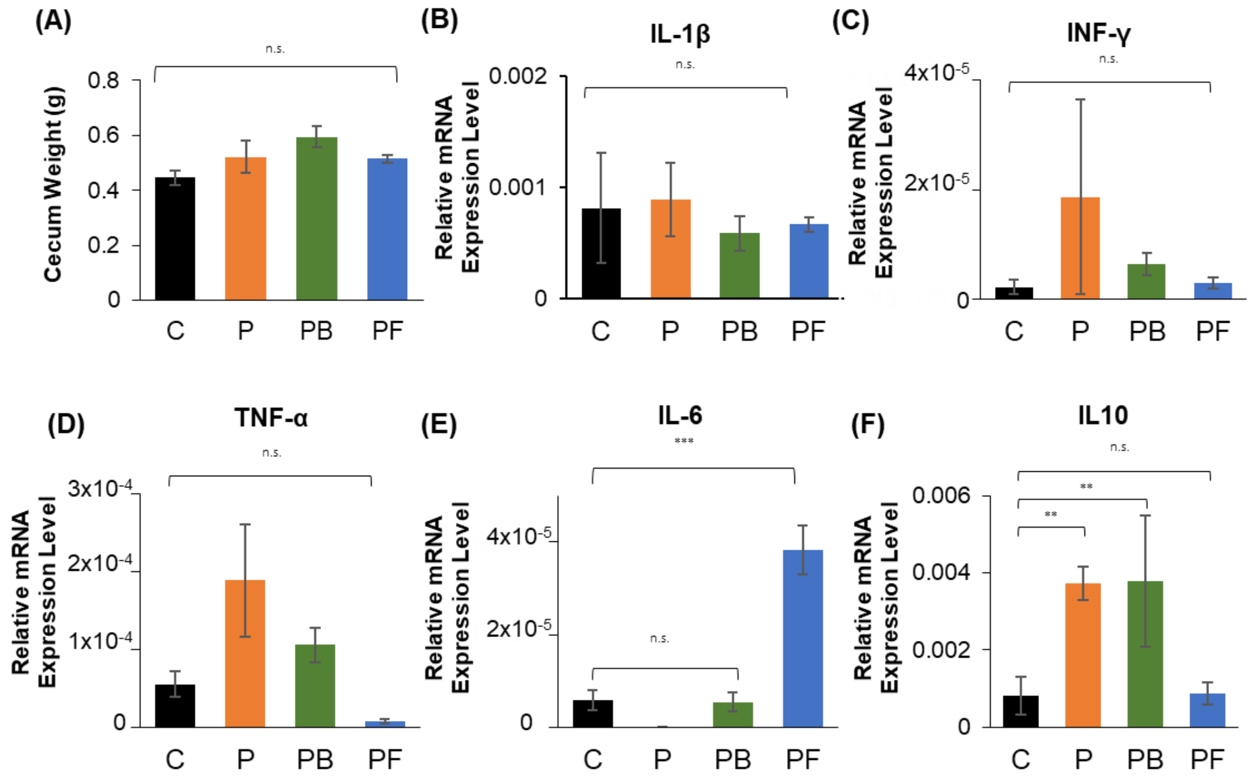

Supplementary Figure 7. Changes in cecum size and expression of inflammation-related genes in ciprofloxacin-treated mice.

At the end of the experiment, we measured cecum weight and measured the mRNA expression levels of inflammation-related genes in the large intestine for ciprofloxacin-treated mice (C: control, P: ciprofloxacin + natural recovey, PB: ciprofloxacin + *B. bifidum*, PF: ciprofloxacin + fecal transplant from control mice). A) Cecum weight, relative mRNA expression of genes encoding B) IL-β, C) TNF-α, D) INF-γ, E) IL-6, and F) IL-10 for ciprofloxacin-treated mice, using *Actb* as a reference gene. Error bars indicate standard error, and significance was determined by One-Way ANOVA and Dunnett's test. (* $p < 0.05$, ** $p < 0.01$, *** $p < 0.001$).
