# Supplementary material for: Bifidobacterium bifidum Suppresses Gut Inflammation Caused by Repeated Antibiotic Disturbance Without Recovering Gut Microbiome Diversity in Mice
Source: Front Microbiol. 2020 Jun 18;11:1349. doi: 10.3389/fmicb.2020.01349 (PMC7314955; doi:10.3389/fmicb.2020.01349)
Supplement: Supplementary file 2 [file Data_Sheet_2.pdf]

Supplementary Material

1 Supplementary Tables

Table S1. Primer sets used for the quantification of inflammation-related gene expression with RT-qPCR

Gene	Forward Primer (5’- 3’)	Reverse Primer (5’- 3’)
Actb	GCTCTTTTCCAGCCTTCCTT	AGGGAGACCAAAGCCTTCAT
Infg	CCTTTGGACCCTCTGACTTG	CGCAATCACAGTCTTGGCTA
Il1b	TGAAATGCCACCTTTTGACA	CTGCCTAATGTCCCCTTGAA
IL-6	CACGGCCTTCCCTACTTCAC	TTCCAAGAAACCATCTGGCTA
IL-10	CCAAGCCTTATCGGAAATGA	CATTCCCAGAGGAATTGCAT
Tnf	CCACATCTCCCTCCAGAAAA	CTCCCTTTGCAGAACTCAGG

Table S2. Two-Way rm-ANOVA results for α-diversity metrics (Shannon and Chao1 Indexes) for natural recovery groups (Control, A, P, and V groups). Two-Way rm-ANOVA was performed to test for statistically significant differences over time (Weeks 0-9).

**Shannon**	Chi-Sq	Df	p value	Significance
Antibiotic-Type	142.7184	3	<2e-16	***
Week	0.1738	1	0.6768	
Antibiotics x Week	3.1144	3	0.3743	
**Chao 1**	Chi-Sq	Df	p value	Significance
Antibiotic-Type	94.9894	3	<2e-16	***
Week	6.0514	1	0.0139	*
Antibiotics x Week	0.3801	3	0.9443	

Significance codes: *** p<0.001, ** p<0.01, * p<0.05

Table S3. Two-Way rm-ANOVA results for based on Bray-Curtis Index of Dissimilarity for natural recovery groups (Control, A, P, and V groups). Two-Way rm-ANOVA was performed to test for statistically significant differences over time (Weeks 0-9).

	Chi-Sq	Df	p value	Significance
Antibiotic-Type	65.9684	3	3.11E-14	***
Week	0.3231	1	0.5698	
Antibiotics x Week	25.8446	3	1.03E-05	***

Significance codes: *** p<0.001, ** p<0.01, * p<0.05

Table S4. Factor loadings for exploratory factor analysis based on the microbial community composition over time (Weeks 0-9) for natural recovery groups (Control, A, P, and V groups). Note that the absolute values for blank entries are less than 0.2, but not zero. The lowest taxonomic rank for which information was available is indicated in square brackets (F: family, G: genus).

	Factor 1	Factor 2	Factor 3
Lactobacillus [G]	0.926644	-0.27507	
Proteus [G]		0.392107	-0.32282
Escherichia coli		0.499274	-0.45402
Desulfovibrionaceae [F]			-0.21764
Bacteroides acidifaciens			0.457251
Bacteroidales S24-7 [F]	-0.24224	-0.64117	-0.50964

Table S5. Two-Way rm-ANOVA results for α-diversity metrics (Shannon and Chao1 Indexes) for vancomycin-treated groups (Control, V, VB, and VF groups). Two-Way rm-ANOVA was performed to test for statistically significant differences over time (Weeks 0-9).

**Shannon**	Chi-Sq	Df	p value	Significance
Treatment	138.5642	3	<2e-16	***
Week	0.3842	1	0.5354	
Treatment x Week	1.0472	3	0.7898	

**Chao1**	Chi-Sq	Df	p value	Significance
Treatment	177.8706	3	<2e-16	***
Week	3.4694	1	0.06251	
Treatment x Week	3.2894	3	0.3743	

Significance codes: *** p<0.001, ** p<0.01, * p<0.05

Table S6. Two-Way rm-ANOVA results for based on Bray-Curtis Index of Dissimilarity for vancomycin-treated groups (Control, V, VB, and VF groups). Two-Way rm-ANOVA was performed to test for statistically significant differences over time (Weeks 0-9).

	Chi-Sq	Df	p value	Significance
Treatment	65.9684	3	3.11E-14	***
Week	0.3231	1	0.5698	
Treatment x Week	25.8446	3	1.03E-05	***

Significance codes: *** p<0.001, ** p<0.01, * p<0.05

Table S7. Factor loadings for exploratory factor analysis of the microbial community composition over time (Weeks 0-9) for vancomycin treated groups (Control, V, VB, and VF groups). Note that the absolute values for blank entries are less than 0.2, but not zero. The lowest taxonomic rank for which information was available is indicated in square brackets (F: family, G: genus).

	Factor 1	Factor 2	Factor 3
Lactobacillus [G]	0.867991	-0.39684	
Proteus [G]		0.655285	0.403186
Escherichia coli		0.356525	0.300518
Bacteroidales S24-7 [F]	-0.40856	-0.51367	0.650558
